# Glucocorticosteroids trigger reactivation of human cytomegalovirus from latently infected myeloid cells and increase the risk for HCMV infection in D+R+ liver transplant patients

**DOI:** 10.1099/vir.0.069872-0

**Published:** 2015-01

**Authors:** Ellen Van Damme, Sarah Sauviller, Betty Lau, Bart Kesteleyn, Paul Griffiths, Andrew Burroughs, Vincent Emery, John Sinclair, Marnix Van Loock

**Affiliations:** 1 Janssen Infectious Diseases BVBA, Turnhoutseweg 30, 2340 Beerse, Belgium; 2 Department of Medicine, University of Cambridge, Level 5, Addenbrooke’s Hospital, Hills Road, Cambridge CB2 0QQ, UK; 3 Division of Infection and Immunity (Royal Free Campus), University College London, Rowland Hill Street, Hampstead, London NW3 2QG, UK; 4 Sheila Sherlock Liver Centre, Royal Free NHS Trust, Hampstead, London NW3 2QG, UK; 5 Department of Microbial and Cellular Sciences, University of Surrey, Guildford, Surrey GU2 7XH, UK

## Abstract

Graft rejection in transplant patients is managed clinically by suppressing T-cell function with immunosuppressive drugs such as prednisolone and methylprednisolone. In such immunocompromised hosts, human cytomegalovirus (HCMV) is an important opportunistic pathogen and can cause severe morbidity and mortality. Currently, the effect of glucocorticosteroids (GCSs) on the HCMV life cycle remains unclear. Previous reports showed enhanced lytic replication of HCMV *in vitro* in the presence of GCSs. In the present study, we explored the implications of steroid exposure on latency and reactivation. We observed a direct effect of several GCSs used in the clinic on the activation of a quiescent viral major immediate-early promoter in stably transfected THP-1 monocytic cells. This activation was prevented by the glucocorticoid receptor (GR) antagonist Ru486 and by shRNA-mediated knockdown of the GR. Consistent with this observation, prednisolone treatment of latently infected primary monocytes resulted in HCMV reactivation. Analysis of the phenotype of these cells showed that treatment with GCSs was correlated with differentiation to an anti-inflammatory macrophage-like cell type. On the basis that these observations may be pertinent to HCMV reactivation in post-transplant settings, we retrospectively evaluated the incidence, viral kinetics and viral load of HCMV in liver transplant patients in the presence or absence of GCS treatment. We observed that combination therapy of baseline prednisolone and augmented methylprednisolone, upon organ rejection, significantly increased the incidence of HCMV infection in the intermediate risk group where donor and recipient are both HCMV seropositive (D+R+) to levels comparable with the high risk D+R− group.

## Introduction

Human cytomegalovirus (HCMV) is a member of the *Betaherpesvirinae* subfamily, which primarily causes morbidity and mortality in neonates or in HIV and transplant patients (Bristow *et al.*, 2011; Husain *et al.*, 2009).

HCMV displays a lytic and a latent phase depending on the infected cell type and the differentiation status of the cell (Bolovan-Fritts *et al.*, 1999; Goodrum *et al.*, 2002; Hahn *et al.*, 1998; Huang *et al.*, 2012; Mendelson *et al.*, 1996; Reeves *et al.*, 2005b; Sindre *et al.*, 1996; Söderberg-Nauclér *et al.*, 1997, 2001; Taylor-Wiedeman *et al.*, 1994; Zhuravskaya *et al.*, 1997). HCMV gene expression is driven by the major immediate-early promoter (MIEP), which is controlled by binding of transcription factors to *cis*-regulatory regions including the enhancer (Cherrington & Mocarski, 1989; Cherrington *et al.*, 1991; Lang & Stamminger, 1993; Macias & Stinski, 1993). During lytic infection, the MIEP is active and the expression of the full array of viral genes results in the production of new virions (Arrode & Davrinche, 2003; Bayer *et al.*, 2013; DuRose *et al.*, 2012; O'Connor & Shenk, 2012; Sinzger *et al.*, 1999a). Latency is established primarily in monocytes and CD34^+^ progenitor cells, although other sites may exist, (Sissons *et al.*, 2002; Springer & Weinberg, 2004) in which the MIEP is suppressed resulting in a limited set of transcripts and the lack of viral progeny (Bego *et al.*, 2005, 2011; Goodrum *et al.*, 2007; Jenkins *et al.*, 2004, 2008; Montag *et al.*, 2011; Reeves, 2011). Differentiation of latently infected cells to dendritic cells (DC) results in de-repression of the MIEP and initiation of the lytic cycle and the release of infectious virus (Reeves *et al.*, 2005a, b, c; Stenberg *et al.*, 1985; Stinski *et al.*, 1983; Wathen & Stinski, 1982).

Currently, HCMV remains an important complication after solid organ transplantation (SOT) despite the pre-emptive or prophylactic use of valganciclovir (Kotton *et al.*, 2013). Symptoms vary but may culminate in organ loss (Boeckh & Nichols, 2004; Crough & Khanna, 2009; Limaye *et al.*, 2006). In SOT, the serostatus of the donor is the most important risk factor to develop HCMV-related disease, especially if the recipient is seronegative (D+R−) (Humar & Snydman, 2009). The initial lack of HCMV-specific immune responses, combined with a suppressed immune response of the recipient may lead to productive HCMV infection of a variety of cell types in the recipient (Arrode & Davrinche, 2003; Bayer *et al.*, 2013; DuRose *et al.*, 2012; O'Connor & Shenk, 2012; Sinzger *et al.*, 1999a). Consequently, the D+R− group is considered a high-risk group. An intermediate risk is found when both the donor and recipient are seropositive (D+R+) and a lower risk in a D−R+ setting where reactivation of latent virus in the recipient in the face of pre-existing immunity occurs. Finally, the lowest risk is when both donor and recipient are HCMV seronegative (Atabani *et al.*, 2012).

The risks for HCMV-related complications post-transplant are often attributed to T-cell suppression by immunosuppressive drugs such as prednisolone or methylprednisolone, both glucocorticosteroids (GCSs), which are used post-transplant in baseline therapies and/or in cases of acute rejection (i.e. augmented therapy) (Bunde *et al.*, 2005; Cope *et al.*, 1997; Cwynarski *et al.*, 2001; Gamadia *et al.*, 2003; Gratama *et al.*, 2001; Hebart *et al.*, 2002; Krause *et al.*, 1997; Ljungman *et al.*, 2011; Nebbia *et al.*, 2007; Ozdemir *et al.*, 2002; Reusser *et al.*, 1991, 1999). The mechanism of action of GCSs is currently not completely understood. GCSs act via the glucocorticoid receptor (GR), which is a ligand-regulated transcription factor (Buckingham, 2006; Pratt *et al.*, 2006; van der Velden, 1998). In the absence of a ligand, the steroid-binding domain of the GR is bound by inhibiting chaperone proteins. Upon GCS binding, GR undergoes a conformational change, releases the chaperones, translocates to the nucleus and binds to a 15 bp glucocorticoid response element (GRE) resulting in transcriptional activation (Buckingham, 2006; Pratt *et al.*, 2006; van der Velden, 1998). However, other mechanisms of GCS that do not involve GR binding to a GRE have been described, these are involved in cytokine production and T-cell inhibition (Almawi *et al.*, 1999; Buckingham, 2006; Paliogianni *et al.*, 1993; Pratt *et al.*, 2006; van der Velden, 1998).

It has also been reported that GCSs have a direct enhancing effect, *in vitro*, on HCMV lytic infection in fibroblasts (St George & Rinaldo, 1994; Tanaka *et al.*, 1984; West *et al.*, 1988) and macrophages (Lathey & Spector, 1991) although one report contradicts these observations (Scott *et al.*, 2000). In contrast, no data are available on the impact of GCSs on reactivation of HCMV from latency. Consequently, we set out to explore the effects of GCSs on reactivation of HCMV from latency. We show that GCSs can activate the MIEP in a GR-dependent pathway and that, through this mechanism prednisolone is able to reactivate latent HCMV from primary monocytes. To investigate the clinical relevance of these findings, we performed a retrospective analysis of a liver transplant population to assess the effect of baseline prednisolone and augmented methylprednisolone in case of acute graft rejection on the incidence, viral kinetics and viral load of HCMV. In case of HCMV reactivation, the peak viral load remains similar between all treatment groups. Treatment with both baseline prednisolone and augmented methylprednisolone significantly increased the incidence of HCMV infection in the D+R+ group where donor and recipient are both HCMV seropositive. Taken together, these data suggest that GCSs play a role in the viral kinetics of latency and reactivation and that treatment with these compounds may increase the probability of HCMV-related complications post-transplantation.

## Results

### GCSs activate the MIEP in a THP-1-MIEP-EGFP reporter assay

We first assessed the influence of GCSs on the activation of the MIEP. In stably transfected THP-1-MIEP-EGFP cells, the MIEP was epigenetically silenced, but was activated upon differentiation of cells by phorbol 12-myristate 13-acetate (PMA; see Table 1). In this robust assay [Z′ of 0.91 (Zhang *et al.*, 1999)], 17 clinically used GCSs and several reference compounds i.e. pam3cys, PMA, ionomycin, SAHA and polyinosinic-polycytidylic acid (Poly I:C) were investigated (Table 1).

**Table 1.** LEC (µM) and maximal response (MaxResp, % increase) of commonly used glucocorticosteroids and reference compounds on MIEP-driven EGFP expression in THP-1 cells (*n* = 3)

Drug name	LEC (µM)	MaxResp (% increase)
Beclometasone dipropionate	0.000060±0.000019	151±37
Budesonide	<0.000039±0.000002	186±34
Dexamethasone	0.000156±0.000036	182±40
Hydrocortisone acetate	0.006167±0.003350	126±35
Methylprednisolone	0.000219±0.000011	159±33
Naflocort	<0.000038±0	196±38
Prednisolone	0.002343±0.000626	151±23
Betamethasone valerate	<0.000038±0	180±29
Desonide	0.000268±0.000105	165±38
Prednisolone acetate	0.003115±0.000485	161±25
Triamcinolone	0.002447±0.003009	125±26
Fluocinolone acetonide	0.000117±0.000076	168±19
Clocortolone pivalate	0.000278±0.000201	138±22
Difluprednate	0.000063±0.000007	153±38
Halcinonide	<0.000038±0	174±18
Alclometasone dipropionate	0.000188±0.000095	148±48
Fluocinonide	0.000138±0.000087	150±29
SAHA	2.070000±0.300000	261±82
Pam3cys	0.000640±0	767±111
PMA	0.000010±0	737±104
Ionomycin	0.082400±0.002200	717±95
Poly I:C	Not active	Not active

All the selected GCSs, as well as reference compounds PMA, SAHA, pam3cys and ionomycin, activated the MIEP whereas Poly I:C did not have any effect. Although SAHA, PMA, ionomycin and pam3cys induced a higher maximal response compared to the GCSs, the GCS-induced increase in MIEP activity was reproducible in 3 independent replicates and over a broad range of lowest effective concentrations (LECs) ranging from nM to fM concentrations.

### GCSs activate the MIEP in a GR-dependent manner in THP-1-MIEP-EGFP cells

**Fig. 1.**
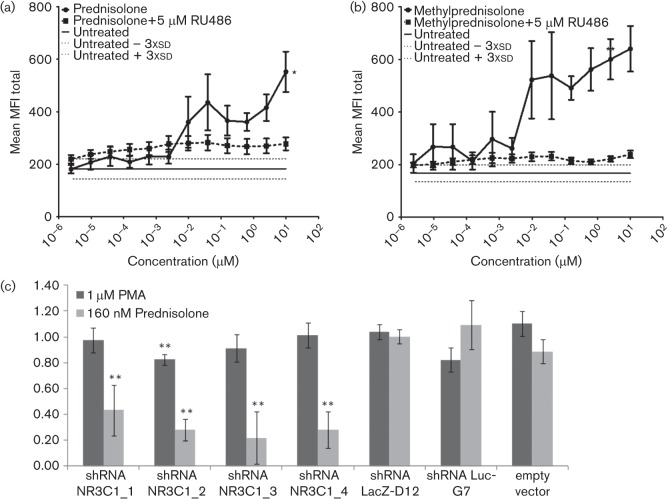
GR-dependent MIEP activation by prednisolone and methylprednisolone. THP-1-MIEP-EGFP cells treated with prednisolone (a) or methylprednisolone (b) in the presence or absence of 5 µM Ru486. Activity of the MIEP was plotted as MFI 24 h post treatment (*n* = 6). The baseline MFI in untreated cells (horizontal full line) and the baseline MFI plus and minus three times the sd (dotted lines) are indicated. Significance was determined using a two-tailed Student’s *t*-test (*P<0.01; **P<0.001). (c) THP-1-MIEP-EGFP cells transfected with shRNA against GR (shRNA NR3C1_1–NR3C1_4), LacZ-D12 and Luc-G7 or empty vector. Cells were treated with 160 nM prednisolone or 1 µM PMA (*n* = 3). Significant decreases in MIEP activation were calculated using a two-tailed Student’s *t*-test (**P<0.001).

**Fig. 2.**
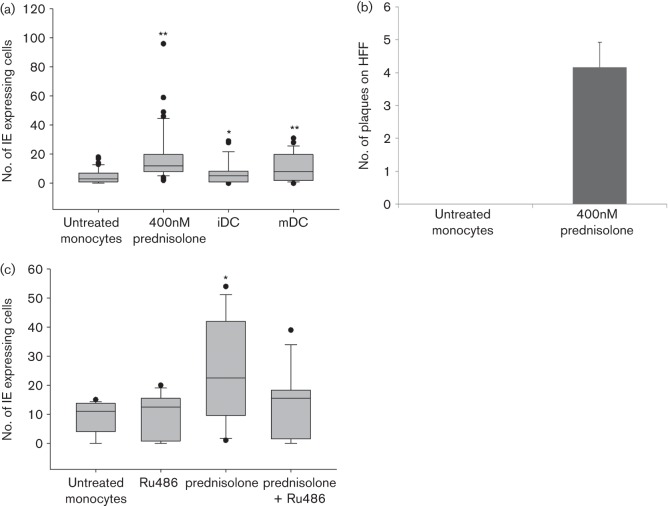
Reactivation of latent HCMV from primary CD14^+^ monocytes by prednisolone. Latently infected (TB40/E or TB40/E-IE2-YFP) CD14^+^ monocytes were treated for 96 h with prednisolone or differentiated to iDCs or mDCs. IE positive cells were manually counted and plotted. (a) Activation of the MIEP in CD14^+^ monocytes by treatment with 400 nM prednisolone or by differentiation to monocyte-derived iDC and mDC (seven independently processed donors). *P* values were calculated by a mixed model corrected for donor dependency (**P<0.0001; *P = 0.001). (b) Treatment of CD14^+^ monocytes with prednisolone results in the production of infectious virus (*n* = 3, three independently processed donors, two technical replicates per donor). (c) Inhibition of activation of the MIEP by Ru486. Latently infected (TB40/E-IE2-YFP) monocytes were left untreated or were treated with 5 µM Ru486, 400 nM prednisolone or the combination of both for 96 h before IE expressing cells were counted manually (five independently processed donors). Using a mixed model corrected for donor dependency, the *P* value was calculated (*P = 0.001). On all boxplots, values outside tenth and ninetieth percentiles were considered outliers (•).

To determine if prednisolone and methylprednisolone activate the MIEP via the GR, THP-1-MIEP-EGFP cells were treated with prednisolone or methylprednisolone in the presence or absence of the GR inhibitor Ru486 (Cadepond *et al.*, 1997). Treatment with prednisolone or methylprednisolone activated the MIEP resulting in increased levels of EGFP expression as determined by mean fluorescent intensity (MFI) of the cell population (Fig. 1a, b). Concentrations of methylprednisolone below 10^−2^ µM did not have any effect and only baseline fluorescence was observed. When GR inhibitor Ru486 was added simultaneously with prednisolone or methylprednisolone, the MIEP could no longer be activated (Fig. 2a, b) suggesting that the GR pathway is followed.

The involvement of GR in GCS-mediated MIEP activation was further demonstrated using THP-1-MIEP-EGFP cells transfected with the empty vector or with shRNAs against the GR, LacZ-D12 and Luc-G7. Analysis using reverse transcriptase quantitative PCR (RT-qPCR) showed that shRNA NR3C1_1, shRNA NR3C1_2, shRNA NR3C1_3 and shRNA NR3C1_4, respectively, knocked down 53.1, 58.5, 17.1 and 29.1 % of the GR expression. None of the negative control shRNAs against LacZ-D12 and Luc-G7 resulted in any knockdown of the GR. Subsequent to shRNA transfection, the cells were treated with 160 nM prednisolone or 1 µM PMA. None of the three negative controls had any effect on PMA or prednisolone-induced MIEP activation. In addition, besides an off-target effect of shRNA NR2C1_2, none of the other GR-specific shRNAs had any effect on MIEP activation with PMA. However, all four shRNAs against GR significantly downregulated the activation of the MIEP after treatment with prednisolone (Fig. 1c).

The activation of the MIEP by methylprednisolone was inhibited by treatment with Ru486 and shRNA knockdown of the GR, strongly suggesting that the MIEP is activated through a GR-mediated mechanism by prednisolone and methylprednisolone.

### Prednisolone reactivates HCMV in primary monocytes

To investigate whether GCSs can mediate reactivation of HCMV from latently infected cells, primary CD14^+^ monocytes were latently infected with wild-type TB40/E or TB40/E-IE2-YFP *in vitro* and subsequently treated with 400 nM prednisolone or differentiated to immature or mature monocyte-derived DCs (iDC and mDC, respectively). Activation of immediate-early (IE) expression was measured by manual counting of IE expressing cells after 96 h (Fig. 2). Despite some variability between donors in the number of IE expressing cells, as reported before (Huang *et al.*, 1996), differentiation to iDCs and mDCs increased the number of IE expressing cells (*P* = 0.001 and *P*<0.0001, respectively). Interestingly, also treatment with prednisolone reproducibly increased IE expression in latently infected monocytes (*P*<0.0001) (Fig. 2a). Although activation of the MIEP is a first step to initiation of the lytic cycle, reactivation of virus from latency necessarily requires the production of infectious virus. Therefore, the supernatant of prednisolone treated and untreated latent monocytes was transferred to fibroblasts and incubated until infectious centres could be observed. Treatment with prednisolone resulted in the production of infectious virus whereas supernatants from untreated, latently infected monocytes did not (Fig. 2b).

**Fig. 3.**
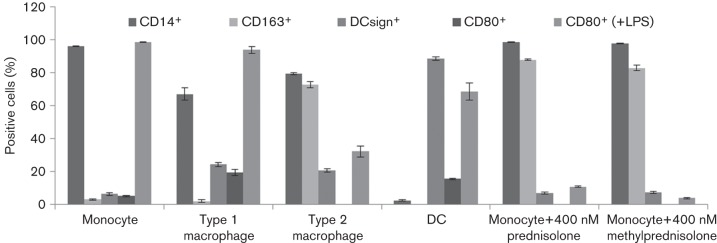
Characterization of the surface marker phenotype of prednisolone -treated monocytes compared with untreated monocytes, type 1 macrophages, type 2 macrophages and DCs. A representative donor is shown (*n* = 3), error bars indicate the variation within donor.

In THP-1 cells, prednisolone activated the MIEP in a dose-dependent manner via the GR. Similarly, we tested reactivation in the presence of the GR inhibitor Ru486 (Fig. 2c) in CD14^+^ monocytes. Ru486 treatment had no effect on levels of IE expression in latently infected untreated CD14^+^ monocytes. As expected, treatment of latently infected cells with prednisolone significantly increased the number of IE expressing cells (*P* = 0.001). We also showed that Ru486 leads to a decrease in number of IE expressing cells when added to prednisolone treated cells (interaction in mixed model, *P* = 0.007). This observation strongly suggests that prednisolone activates the HCMV MIEP via a GR-mediated pathway. While the best described mechanism of GCS-induced cellular events is through GR binding to a GRE consensus site, we did not detect any reproducible interaction between the MIEP and the GR despite repeated chromatin immunoprecipitation (ChIP) experiments (data not shown). Alternatively, prednisolone may trigger MIEP activation by inducing differentiation of monocytes to terminally differentiated macrophages or DCs. Consequently, we tested if prednisolone changed the differentiation state of monocytes (Fig. 3). Using a cluster of differentiation markers specific for monocytes [CD14^+^/CD163^−/lo^/DCsign^−^/CD80^+^ upon lipopolysaccharide (LPS) activation], type 1 macrophages (CD14^+^/CD163^−^/DCsign^lo^/CD80^+^ upon LPS activation), type 2 macrophages (CD14^+^/CD163^+^/DCsign^lo^/CD80^lo^ upon LPS activation) and dendritic cells (CD14^−^/CD163^−^/DCsign^high^/CD80^+^ upon LPS activation), we showed that prednisolone treatment did not result in differentiation of monocytes to DCs or type 1 macrophages, but did increase markers of type 2 macrophages (e.g. CD163, absent response in CD80 to LPS).

Taken together, these data suggest that prednisolone can reactivate HCMV from monocytes resulting in the production of viral progeny. The mechanism via which prednisolone activates the HCMV MIEP, and likely subsequent reactivation, appears to be GR-driven, however, it is unlikely to proceed via binding of the GR to the MIEP and probably occurs via an alternative pathway. In addition, prednisolone leads to the induction of a subset of macrophage type 2-like cell type markers suggesting a novel differentiation-induced mechanism for HCMV reactivation by GCSs.

### The use of GCSs increases the risk for HCMV infection in the D+R+ group of liver transplant recipients.

In order to mirror the *in vitro* experiments, we investigated the *in vivo* effects of steroids on the incidence of HCMV infection, the viral load and the timing of infection in liver transplant recipients in the context of the HCMV serostatus of donor and recipient. Patients were divided into four categories depending on the receipt of prednisolone for baseline immunosuppression and/or methylprednisolone for augmented immunosuppression. There were no statistically significant differences in the incidence of HCMV DNAemia in any of the steroid subgroups or in the peak or cumulative HCMV loads in patients within the subset of viraemic patients based on receipt of steroids as baseline or augmented immunosuppression (Table 2). Viral loads were highest in the patients receiving no steroids, but were comparable in the other three groups. In the context of the timing of the occurrence of HCMV DNAemia (defined as a VL in whole blood >200 genomes ml^−1^) patients who received augmented steroid immunosuppression, irrespective of whether they had baseline prednisolone, had a shorter time to DNAemia (21.5 days) compared to the other two groups (no steroid treatment or prednisolone maintenance only) (*P* = 0.05; Table 2).

**Table 2.** Effects of different combinations of baseline maintenance steroid immunosuppression and augmented steroid immunosuppression. Given are the days to DNAemia and peak HCMV load in blood. Statistically significant values are indicated (*)

Maintenance prednisolone (Yes/No)	Augmented methylprednisolone (Yes/No)	Incidence of DNAemia (no. with DNAemia/ total in group)	Peak HCMV load [mean of log10 genomes ml^−1^ (range)]	Days to DNAemia [mean no. of days (range)]	
No	No	24/92	3.47 (2.39–5.08)	25 (1–56)	
Yes	No	17/73	3.38 (2.36–5.12)	29 (1–57)	
No	Yes	21/72	2.96 (2.40–4.75)	23 (5–85)	
Yes	Yes	23/64	3.49 (2.58–4.91)	21* (1–53)	

**Fig. 4.**
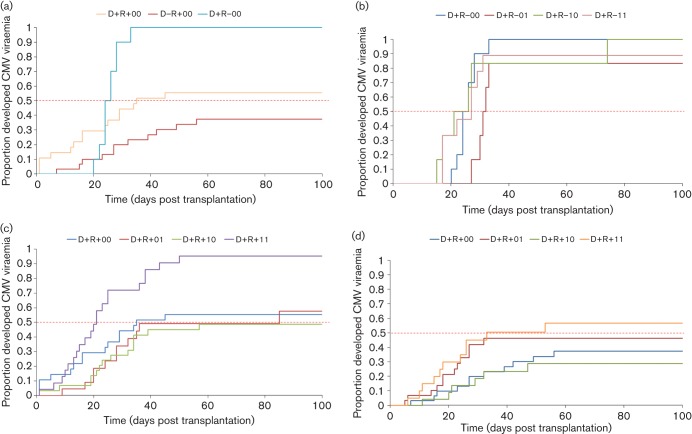
Kaplan–Meier curves showing time to occurrence of DNAemia of the different donor-recipient serogroups stratified according to GCS use. (a) Overview of the three risk groups for HCMV-related complications (untreated). The different subgroups i.e. D+R+ (b), D+R− (c) and D−R+ (d) were evaluated under different immune-suppressive regimens. 0.0, Regimen not based on steroids; 0.1, baseline therapy non-steroid plus augmented methylprednisolone treatment; 1.0, baseline prednisolone treatment; 1.1, baseline prednisolone treatment plus augmented methylprednisolone treatment.

Further, we investigated the incidence of DNAemia in different donor-recipient groups according to steroid use. As published previously, the highest risk for HCMV-related complications was when a seropositive organ is introduced in a seronegative recipient (D+R−) (Humar & Snydman, 2009). The lowest chance of developing HCMV disease was when the recipient was seropositive for HCMV but faced reactivation of HCMV that was present already in the body upon immunosuppression (D−R+) (Fig. 4a). In the D+R− group of liver transplant recipients, the incidence of DNAemia was very high (>90 %) and therefore the impact of baseline and augmented steroid use on virological parameters was difficult to assess and no significant differences were observed (Fig. 4, Table 3). However, in the D+R+ and D−R+ groups the lower incidence of DNAemia provides an opportunity to assess the impact of steroid use on the incidence of HCMV DNAemia. In the D−R+ group, DNAemia rates were comparable between patients who received no steroids and those receiving maintenance steroids and augmented steroids. Patients who received steroids in both clinical settings showed a non-significant increase in the incidence of HCMV DNAemia. The difference in DNAemia between the group with maintenance steroids and maintenance plus augmented steroids was of borderline significance (*P* = 0.07). In contrast, in the D+R+ group the incidence of HCMV DNAemia in patients receiving both baseline steroids and augmented steroids compared to the other groups was more pronounced. Statistically significant increases in the incidence of HCMV DNAemia in the steroid -treated patients (baseline and augmented) were observed compared to all other groups (as individual comparisons; Fig. 4, Table 3).

**Table 3.** Incidence of HCMV DNAemia in the different D/R serogroups for HCMV based on maintenance and augmented steroid use

Maintenance/augmented steroids (1 = Yes, 0 = No)	D+R− (*n* = 30)	D−R+ (*n* = 110)	D+R+ (*n* = 104)
0.0	10/11	12/35	15/28
0.1	5/5	13/30	11/22
			
1.0	6/6	6/24	15/30
1.1	8/9	11/21	22/24*

* Significantly different to the 0.0 group (*P* = 0.005), the 1.0 group (*P* = 0.001) and the 0.1 group (*P* = 0.003).

Under the influence of GCSs, the D+R+ group had an increased risk of developing HCMV DNAemia shifting them from an intermediate risk group to a high risk group, which is comparable to the D+R− group.

## Discussion

HCMV-related illness is an important risk factor post-transplant and an increased incidence of HCMV in transplant settings has been associated with GCS treatment (Cope *et al.*, 1997; Nebbia *et al.*, 2007). Nevertheless, the effects of GCSs on the virus itself have not been fully investigated, especially the influence of GCSs on latency and reactivation. As de-repression of the MIEP is a crucial step in the switch between lytic and latent infection (Ghazal *et al.*, 1990; Lubon *et al.*, 1989; Reeves, 2011; Sinclair, 2010), we used a THP-1-MIEP-EGFP reporter assay to assess the MIEP activating potential of GCSs. THP-1 cells have been used previously to study latency and reactivation of HCMV and, consistent with the known silencing of the MIEP in these cells after infection, MIEP-driven EGFP expression was suppressed in the absence of a stimulus (Ioudinkova *et al.*, 2006; Keyes *et al.*, 2012). However, treatment of the THP-1-MIEP-EGFP cells with 17 clinically relevant GCSs, as well as several reference compounds known to activate the MIEP (SAHA, ionomycin and PMA) (Groves *et al.*, 2009; Phillips *et al.*, 2005; Weinshenker *et al.*, 1988); to induce transcription factors, such as NFκB and AP-1, associated with MIEP activation (Pam3cys) (Parker *et al.*, 2004; Pevsner-Fischer *et al.*, 2007; Reeves, 2011) or compounds known to reactivate other latent viruses (SAHA) or HCMV (PMA) (Archin *et al.*, 2012; Keyes *et al.*, 2012) resulted in the activation of the MIEP. Despite the presence of Toll-like receptor 3 in THP-1 cells, immune-regulator Poly I:C did not activate the MIEP (Alexopoulou *et al.*, 2001; Kumar *et al.*, 2006).

The major pathway through which GCSs exert their effect is the GR pathway. In fact, treatment with small molecule GR inhibitor Ru486 (Cadepond *et al.*, 1997) or shRNA-mediated knock-down of GR completely abolished activation of the MIEP by prednisolone or methylprednisolone. In contrast, neither Ru486 nor shRNA against GR had any effect on PMA-mediated activation of the MIEP, this suggests the involvement of the GR pathway in GCS-mediated MIEP activation.

In addition, a CD14^+^ monocyte latency model was used to investigate if GCSs could activate the MIEP and induce full reactivation of latent virus. In untreated infected CD14^+^ monocytes, a small number of IE expressing cells were observed, but there was no evidence of full reactivation of virus in these cells. Similar observations have been made in models of MCMV latency (Kurz *et al.*, 1999), and we think it is likely that in some monocytes IE gene expression occurs sporadically but without progression to productive infection. This is consistent with the view that, whilst IE1 gene expression is essential for initiation of reactivation of virus, it may not always be sufficient, and other checkpoints have to be passed before full reactivation leading to infectious virus release occurs. In contrast, treatment with prednisolone resulted in a GR-dependent reactivation of IE gene expression and the production of infectious virus. Although robust, this reactivation of infectious virus was at low levels, which is consistent with mouse models of reactivation from latency which have also shown that only a few IE1 transcription reactivation events appear to result in productive reactivation (Kurz & Reddehase, 1999). Interestingly, treatment with GCSs induces an anti-inflammatory state in monocytes and macrophages (Joyce *et al.*, 1997) and hydrocortisone treatment of monocytes induces an anti-inflammatory cell type resembling a type 2 macrophage (Ehrchen *et al.*, 2007), which are known to be permissive for HCMV infection *in vitro* (Bayer *et al.*, 2013). Our data show that, unlike hydrocortisone which appears to only induce IE expression in natural latent monocytes (Taylor-Wiedeman *et al.*, 1994), prednisolone is capable of fully reactivating latent HCMV in experimentally latent monocytes and this is likely due to the ability of prednisolone to induce a strongly anti-inflammatory macrophage type 2-like cell phenotype. Repeated attempts to detect binding of the GR to the MIEP by ChIP in myeloid cells have not, so far, been successful, perhaps suggesting an indirect mechanism. It is worth emphasizing that GCSs have also been shown to regulate gene expression via other mechanisms, for instance via modulation of mRNA stability or via protein–protein interactions (Buckingham, 2006; Pratt *et al.*, 2006; van der Velden, 1998).

It is well established that HCMV contributes to post-transplant morbidity; HCMV may cause damage to the transplanted organ, increases the risk for graft rejection and may cause HCMV syndrome (Crough & Khanna, 2009; Ljungman *et al.*, 2011; Volpin *et al.*, 2002). To address whether our *in vitro* findings had implications *in vivo*, we retrospectively investigated a cohort of liver transplant patients who had received GCSs as immune-suppressants. In the overall patient population, we detected an earlier onset of HCMV DNAemia due to the combination therapy of baseline and augmented steroids. Once the patients went from seronegative to seropositive and thus showed HCMV reactivation, the peak viral load was similar in all treatment groups regardless of steroid use. No difference was observed in the incidence of DNAemia, this is probably attributed to the very high incidence of HCMV in the D+R− group. This is clarified when assessing the individual risk groups. In the absence of GCSs, HCMV DNAemia was more prevalent in the D+R− group such that by day 30, 90 % of D+R− patients had experienced DNAemia compared to 45 % of the D+R+ group and 20 % of the D−R+ group. The high incidence of DNAemia in the D+R− group precluded an assessment of the impact of GCS; however, the other donor-recipient serogroups were amenable for study. In the D−R+ group, GCSs will only affect latent HCMV in the recipient. Treatment of patients in the D−R+ group with both baseline and augmented steroid therapy increased the incidence of HCMV DNAemia. However, the limited number of patients did not allow the study to reach statistical significance (*P* = 0.07) at this point. In contrast, in the D+R+ group, a significant increase in DNAemia incidence was observed in the same treatment regimen. In this group, GCSs can induce reactivation of latent HCMV transferred with the transplanted organ in addition to latent HCMV already present in the recipient. In the Kaplan–Meier time to DNAemia curves, the combination of both maintenance and augmented steroids led to both the earlier detection of DNAemia and increased likelihood of occurrence in the D+R+ group.

Taken together, the *in vitro* and clinical data presented here strongly suggest the need to further investigate the influence of GCSs on HCMV biology during *in vitro* and *in vivo* studies.

## Methods

### 

#### Cell lines and primary cell types.

ARPE-19 cells (CRL-2302; ATCC) and neonatal NHDF fibroblasts (CC-2509; Lonza) were propagated respectively in Dulbecco’s modified Eagle’s medium, nutrient mixture F-12 (DMEM/F12) with l-glutamine (733-1713; BioWhittaker) and Minimal essential medium (MEM), both containing 10 % heat inactivated-FCS (HI-FCS) and 0.04 % gentamicin. THP-1 cells (TIB-202; ATCC) and THP-1-MIEP-EGFP (see below) were grown in RPMI (Lonza) supplemented with 10 % HI-FCS and 0.04 % gentamicin. HEK293 cells were cultured in DMEM (Lonza) containing 10 % HI-FCS, 0.02 mg gentamicin ml^−1^ and 1 % l-glutamine.

Primary monocytes were differentiated to DCs, type 1 macrophages and type 2 macrophages in X-Vivo-15 medium containing 2.5 mM l-glutamine supplemented with 100 ng GMCSF ml^−1^ and 100 ng IL-4 ml^−1^, adapted from Hargett & Shenk (2010) and Verreck *et al.* (2006), 100 ng GMCSF ml^−1^ or 100 ng MCSF ml^−1^, derived from Verreck *et al.* (2006), respectively, for 7 days (all from Peprotech).

#### Viruses.

High endothelial tropic stocks of the HCMV TB40/E (kindly provided by C. Sinzger; Sinzger *et al.*, 1999b), TB40/E-IE2-YFP (Straschewski *et al.*, 2010) and TB40/E-IE2-EGFP (kindly provided by Professor Dr M. Winkler) strains were generated on ARPE-19 cells. ARPE-19 cells were seeded in 175 cm^2^ flasks at 40–60 % confluency 24 h prior to infection with TB40/E at m.o.i. 0.02 (3 h, room temperature). The supernatant was collected until full cytopathic effect. The supernatant of each harvest was spun down for 10 min at 600 ***g***. The best six harvests were used for virus concentration by centrifugation (1 h at 3000 ***g***) and ultracentrifugation (1 h at 5800 ***g***). Ultracentrifuged virus was resuspended in medium and kept at −80 °C. The viral titre was determined by immunofluorescence staining of the IE antigen [anti-I.E.A. (11-003); Argene and Alexa Fluor 488 goat anti-mouse IgG (A-11001); Life Technologies].

#### MIEP-EGFP reporter THP-1 cell line.

The MIEP of Merlin was amplified using Phusion High Fidelity polymerase (New England Biolabs) according to the manufacturer’s instructions using 0.3 mM forward (TTCAAAATTTTATCGGATTTCTGTCGCCGACTAAATTCA) and reverse (CTGGACTAGTGGATCCGGTGTCTTCTATGGAGGTCA) primer. Subsequently, the HCMV promoter of pLenti6.3-EGFP was removed by a *Cla*I/*Bam*HI digest (Roche) and after vector dephosphorylation (TSAP; Promega), the MIEP PCR fragment was cloned in pLenti6.3-EGFP.

A stable cell line was established by lentiviral transduction. MIEP-EGFP-lentivirus was prepared by seeding 2×10^6^ HEK293 cells in 15 cm^2^ Petri dishes. Two days later, the cells were triple lipo-transfected with 5 µg pLenti-MIEP-EGFP, 5 µg GAG-pol packaging plasmid and 2 µg VSVG envelope plasmid (lipofectamine; Life Technologies). Twenty-four hours post-transfection, the medium was replaced with 10 ml fresh medium supplemented with 1 mM sodium butyrate. The next day, the medium was spun down at 600 ***g*** for 10 min and stored at −80 °C. THP-1 cells were seeded and stably transduced with the MIEP-EGFP lentiviral vector. The MIEP was stimulated with 1 µM Pam3cys for 24 h after which the EGFP positive cells were sorted using a FACS Aria cell sorter (BD). The cells were incubated and grown for 2 weeks after transfection until the MIEP was silenced again to background fluorescence.

#### THP-1-MIEP-EGFP reporter assay.

The assay was validated to ensure high robustness (Z′ = 0.91) (Zhang *et al.*, 1999). In brief, 7.5×10^4^ cells were seeded in a 96-well plate in the presence of a fourfold dilution series of a compound. Twenty-four hours after compound addition, the percentage of EGFP expressing cells and the MFI was read on a FACS Canto II. For each tested compound, the lowest effective concentration (LEC) was determined i.e. the minimal concentration needed to lift the restriction of the MIEP and to produce a fluorescent signal three standard deviations above the median of the background signal. In addition, the maximal response (MaxResp) was determined i.e. the increase in signal compared to the background where 100 % increase equals a doubling in the percentage of cells with an active MIEP.

#### shRNA against GR.

shRNAs directed against the GR receptor (NR3C1 gene, TRCN0000245003-6), control genes LacZ (TRCN0000072225) and Luc (TRCN0000072256), and the empty vector (TRCN0000208001) were found on the RNAi Consortium shRNA Library website. Using these sequences, the appropriate lentivirus was ordered (Sigma Aldrich).

In brief, 10^5^ shRNA transduced THP-1-MIEP-EGFP cells were seeded in a 96-well plate. The MIEP was activated with 160 nM prednisolone, 1 µM PMA or left quiescent (DMSO). Twenty-four hours later, the percentage of EGFP expressing cells was determined using a FACS Canto II (BD).

An average of all the negative control shRNAs was calculated and used to normalize the other measurements and to calculate the fold decrease. A two-tailed Student’s *t*-test was used to determine *P* values. All *P* values below 0.001 were considered significant decreases of MIEP activation.

The percentage of GR knockdown by the various shRNAs was evaluated using RT-qPCR. THP-1-MIEP-EGFP cells transduced with the shRNA were used to prepare RNA samples (RNeasy kit; Qiagen) following the manufacturer’s instructions. cDNA was prepared using Superscript-III reverse transcriptase (Life Technologies). RT-qPCR analysis was performed using a qPCR core kit (Eurogentec) with primer and probe sets for NR3C1 (GR receptor, Hs00353740_m1), PGK1 (Phosphoglycerate kinase 1, Hs99999906_m1) and TFRC (transferring receptor CD71, Hs00174609_m1) (Applied Biosystems) according to the manufacturer’s instructions in an AB7900HT Sequence analyser (Applied Biosystems).

#### Primary monocyte latency model.

PBMCs and primary monocytes were isolated from whole blood obtained via the Antwerp Blood Transfusion Center.

In brief, 50 ml blood was diluted in 100 ml ice-cold PBS and applied on 15 ml ficoll-paque layer. The PBMC fraction was separated by centrifugation (800 ***g*** for 20 min, no brake), collected and washed three times using ice-cold PBS. Monocytes were isolated using magnetic CD14 microbeads (Miltenyi) as instructed by the manufacturer. The monocytes were seeded at 10^5^ cells per well (96-well plate, tissue culture treated; Costar) and allowed to adhere for 3 h at 37 °C and 5 % CO_2_. After attachment, the PBS was removed and 200 µl X-Vivo-15 medium (Lonza) was added. After overnight incubation at 37 °C and 5 % CO_2_, the cells were infected with TB40/E-IE-YFP or TB40/E at m.o.i. 5 (3 h at room temperature on a rocking platform). After infection, the virus was removed and 200 µl fresh X-Vivo 15 (2.5 mM l-glutamine) was added. The cells were incubated for 3 days before adding compounds or before they were differentiated. Fluorescent cells were counted manually after 96 h.

Treatment effects on cell count were tested while correcting for donor dependency in a mixed model. Cell counts were log-transformed to approach normality and pairwise comparisons were corrected for multiple testing with the Tukey procedure. The mixed model analysis was performed in SAS 9.2 (Littell *et al.*, 2006).

#### Retrospective analysis of liver transplant patients.

All liver transplants (374 patients) performed and/or followed up at the Royal Free Hospital, London, between July 2002 and the end of January 2010 were identified from the transplant database. All patients gave their written informed consent for their laboratory results to be analysed for research purposes. The studies and consent procedures were approved by the Local Research Ethics Committee of the Royal Free Hospital, London.

Patients were excluded if their HCMV serostatus, or that of their donor, was unknown, if they had participated in the active arms of other ongoing studies including an experimental HCMV vaccine study reported elsewhere (Griffiths *et al.*, 2011), if they had received valganciclovir prophylaxis or if they received a multi-organ transplant. Using these criteria, 321 liver recipients (108 females and 213 male, ages 19–83, mean age 48.9 years) were included in the analysis.

As baseline therapy in liver transplant patients, tacrolimus (Prograf; Fujisawa) at 0.1 mg tacrolimus kg^−1^ day^−1^ was given nasogastrically in two divided doses and started within 6 h after transplantation. Azathioprine was given intravenously and then orally (1 mg azathioprine kg^−1^ day^−1^), and methylprednisolone (16 mg methylprednisolone day^−1^ intravenously) was given until oral intake was possible; then, 20 mg prednisolone day^−1^ was used. Tacrolimus dosing was evaluated every other day and was adjusted with the goal of maintaining a whole blood level between 5–10 ng tacrolimus ml^−1^. The azathioprine dose was not changed unless neutropenia developed. Prednisolone was gradually tapered from 3 weeks and then stopped between 3 and 6 months.

Acute cellular rejection episodes after liver transplantation were managed with augmented steroid therapy using 1 g methylprednisolone day^−1^ for 3 days and repeated if rejection continued (up to a maximum of 12 g in total) in addition to the baseline immunosuppression. If HCMV DNAemia was detected in a liver transplant recipient, no changes in immunosuppressive therapy were undertaken initially. However, depending on immunological risk (i.e. D+R−) and/or high levels of HCMV load tacrolimus levels were reduced at the discretion of the treating physician.

In the context of patients with baseline immunosuppressive steroid and augmented immunosuppressive steroid data a total of 301 patients were available for analysis.

The HCMV IgG serostatus of all patients pre-transplant was determined using the bioMérieux VIDAS assay until July 2008 after which the Abbott Architect I2000 SR assay was used. Donor HCMV IgG serostatus was determined using the same methods or, in the case of donors from other hospitals, was provided by the National Health Service British Transplant Service.

HCMV DNA in whole blood was quantified using a real-time PCR approach described elsewhere (Atabani *et al.*, 2012). HCMV DNAemia was defined as detection of HCMV DNA in whole blood above the assay cut-off (200 genomes ml^−1^). Whole blood samples for HCMV surveillance were collected twice a week while patients remained in hospital and as out-patients for the first 60 days post-transplant, then once a week with a targeted minimum follow-up of the first 90 days after transplantation. Additional samples were collected from HCMV viraemic patients to follow episodes through to PCR-negativity. In patients with HCMV DNAemia surveillance occurred over 149 days (range 5–415 days) and for those without DNAemia, 94.9 days (range 0–247 days).

For comparisons of frequency of DNAemia Fisher’s exact test was used. The time to onset of DNAemia (not normally distributed), the medians were analysed in a Mann–Whitney test. Kaplan–Meier survival analysis was performed to assess time to event (DNAemia onset) in the different steroid subgroups. In all analysis a *P* value ≤0.05 was regarded as significant.
